# The Correlation between Adolescent Daily Breakfast Consumption and Socio-Demographic: Trends in 23 European Countries Participating in the Health Behaviour in School-Aged Children Study (2002–2018)

**DOI:** 10.3390/nu15112453

**Published:** 2023-05-24

**Authors:** Giacomo Lazzeri, Silvia Ciardullo, Angela Spinelli, Daniela Pierannunzio, Anna Dzielska, Colette Kelly, Einar B. Thorsteinsson, Gentiana Qirjako, Anouk Geraets, Kristiina Ojala, Manon Rouche, Paola Nardone

**Affiliations:** 1 Department of Molecular and Developmental Medicine, University of Siena, 53100 Siena, Italy; 2 National Centre for Disease Prevention and Health Promotion, Italian National Institute of Health, 00161 Rome, Italy; 3 Department of Child and Adolescent Health, Institute of Mother and Child, 01-211 Warsaw, Poland; 4 Health Promotion Research Centre, University of Galway, H91 TK33 Galway, Ireland; 5 Einar B. Thorsteinsson School of Psychology, University of New England, Armidale, NSW 2351, Australia; 6 Faculty of Medicine, University of Medicine, 8RRM+W7X Tirana, Albania; 7 Department of Social Sciences, University of Luxembourg, L-4365 Esch-sur-Alzette, Luxembourg; anouk.geraets@uni.lu; 8 Research Center for Health Promotion, Faculty of Sport and Health Sciences, University of Jyvaskyla, FI-40014 Jyvaskyla, Finland; kristiina.ojala@jyu.fi; 9 Research Centre in Epidemiology, Biostatistics and Clinical Research, School of Public Health, Université Libre de Bruxelles, CP598 Brussels, Belgium

**Keywords:** adolescents, breakfast, HBSC, daily breakfast consumption, family affluence, family structure, cross-time data, cross-national data, SES, trends, Europe

## Abstract

Breakfast is often considered the most important meal of the day and can benefit adolescent health in several ways. The aims of the present study were (1) to identify adolescents’ socio-demographic (sex, family affluence and family structure) determinants of daily breakfast consumption (DBC) and (2) to describe trends in DBC among adolescents across 23 countries. Cross-sectional surveys of nationally representative samples of adolescents (aged 11, 13, and 15 years) (*n* = 589,737) participating in the Health Behaviour in School-aged Children (HBSC) survey from 2002 to 2018 were used. Multilevel logistic regression analyses modeled DBC over time, adjusted for family affluence, family structure and year of survey. Four countries showed an increased trend in DBC (the Netherlands, Macedonia, Slovenia, and England). A significant decrease in DBC was observed in 15 countries (Belgium-Fr, France, Germany, Croatia, Portugal, Spain, Hungary, Poland, Russian Federation, Ukraine, Denmark, Finland, Latvia, Lithuania and Sweden). In 4 countries no significant change was observed (Czech Republic, Scotland, Ireland and Norway). In most of the countries (*n* = 19), DBC was higher among the adolescents from high-affluence homes. In all the countries analysed, the adolescents living in two-parent households report higher DBC use than those in single-parent households. More than half of the countries showed a decrease in DBC. There is a need to implement key interventions by developing different strategies (education, incorporating educational curriculum and counselling programmes) to increase DBC. Comparing DBC patterns across HBSC countries is important for understanding regional and global trends, monitoring strategies, and developing health promotion programmes.

## 1. Introduction

A key component of a healthy diet is breakfast. Most age groups consume breakfast regularly, except for adolescents. This fact warrants special attention [1]. Efforts have focused specifically on promoting breakfast intake among schoolchildren due to the associated benefits. Indeed, breakfast consumption among children and adolescents is inversely related to body mass index (BMI) and overweight in both cross-sectional [2,3,4,5] and longitudinal studies [6,7]. Habitual breakfast consumption has been associated with a higher dietary quality and nutrient profiles in children [5,6,8], with improved cognitive performance [4,9,10,11,12,13], and better mental health [14]. Eating breakfast is believed to reduce snacking and the consumption of energy-rich foods of poor nutrient density [1,4,5,15]. In addition, the practice of a regular and healthy breakfast in childhood may persist into adulthood [16,17,18].

Despite its benefits, large surveys have shown that many children and adolescents do not eat breakfast regularly. Findings from the 2018 Health Behaviour in School-aged Children (HBSC) survey showed a significant difference between boys’ and girls’ daily breakfast consumption (DBC) among 11-, 13- and 15-year-old European adolescents, with DBC at 61% for boys and 55% for girls [19]. Data from the United States’ 2015–2018 National Health and Nutrition Examination Survey (NHANES) highlighted the fact that 14% of 6–11-year-olds and 27% of 12–18-year-olds in the did not eat breakfast [20]. In addition, other studies examining trends over time reported that breakfast skipping among adolescents has increased over the past few decades in the United States [21,22]. To our knowledge, only one study has examined trends in breakfast consumption over time (from 2002 to 2010) across multiple countries using the same standardized methods of data collection, with nationally representative samples [23].

Numerous factors may influence breakfast consumption, including socio-economic status (SES), family structure, ethnic origin and sex. In particular, being an adolescent in a family of low SES is associated with irregular breakfast habits. This relationship exists for a range of different SES indicators, such as parental education [24], parental occupation [2,3], family affluence [4] and area-level economic indicators [5,6]. Moreover, while an overall increase in DBC was found in the Scottish HBSC study [25], a decrease in DBC was observed between 1994 and 2010 for older children and those from single-parent families in the United States [24]; other studies showed higher DBC among children from two-parent families [6,7,8,9,10,11,12,13]. Findings from the international 2018 HBSC report and other studies showed that DBC was higher among boys than girls [19,26]. Information on social and economic determinants of DBC among children and adolescents is important for identifying young people and families in need of intervention and for planning initiatives that encourage DBC.

The aims of this study are (i) to identify socio-demographic (sex, family affluence, and family structure) determinants of DBC and (ii) to analyze trends in DBC from 2002 to 2018 in adolescents aged 11, 13 and 15 who come from the 23 countries participating in the HBSC survey. This study contributes to the existing literature by being the first study to compare the DBC trend data of 23 European countries while using the same protocol for two decades.

## 2. Materials and Methods

Data were obtained from five HBSC surveys, conducted in 2001/02, 2005/06, 2009/10, 2013/14 and 2017/18. The HBSC study is a World Health Organization (WHO) collaborative study involving an international network of research teams across Europe and North America. The main purpose of the HBSC study is to gather information on the health and health-related attitudes of adolescents. The target populations chosen for sampling are 11-, 13-, and 15-year-old students [27]. Every country uses a standardized international research protocol to ensure the consistency of the survey methods, data collection and processing. More detailed information about the study is available elsewhere [27,28,29].

An internationally standardized self-report questionnaire was administered in the classroom by trained personnel, teachers or school nurses. The data collection was anonymous, and no directly identifiable information on individual pupils was collected. The survey administrators in each country received an ethics approval from an appropriate regulatory body, and informed consent was obtained from the participants and a parent or guardian [27].

The information from the 23 countries was included in the analyses. The countries were grouped according to the International Country Codes [30].

### 2.1. Measures

To measure DBC, the students were requested to report how many days they usually ate breakfast (defined as more than one glass of milk or juice) on school days and during weekends, respectively. The categories of responses were ‘I never have breakfast during the week’, ‘One day’, ‘Two days’, ‘Three days’, ‘Four days’ and ‘Five days’ for schooldays; and ‘I never have breakfast during the weekend’, ‘I usually have breakfast on only one day of the weekend (Saturday OR Sunday)’ and ‘I usually have breakfast on both weekend days (Saturday AND Sunday)’. These responses were summed to a total range of days of eating breakfast (0–7 days a week) and dichotomized into DBC (7 days) and non-DBC (0–6 days).

### 2.2. Explanatory Variables

Socio-economic position: the Family Affluence Scale (FAS), a validated proxy measure of material affluence [31], was applied to evaluate students’ socioeconomic status. The total score was constructed from the following four items: ‘Does your family own a car, van or truck?’ (No/Yes, one/Yes, two or more (0–2 points)); ‘Do you have a bedroom to yourself?’ (No/Yes (0–1 points)); ‘In the past twelve months, how often have you gone on vacation (vacations) with your family?’ (Not at all/Once/Twice/More than twice (0–3 points)); and ‘How many computers does your family own?’ (None/One/Two/More than two (0–3 points)). The FAS score was divided into low (0–3 points), medium (4–6 points) and high (7–9 points). In 2018, the HBSC group adopted a FAS update (i.e., FAS III). In order to compare the data from the 2002–2018 surveys and perform the trend analysis, the previous version of FAS (i.e., FAS II) has been used in this study.

Family structure: Based on student reports on who they live with most of the time, family structure was categorized as ‘living with two parents’, ‘one parent’ or ‘others’.

### 2.3. Statistical Analyses

A descriptive and trend analysis was made using the data from the 23 countries included in the HBSC surveys, from 2002 to 2018. Only the countries with data on DBC in all five waves were considered in this study. Multilevel logistic regression analyses for the binary outcome variable DBC were conducted for each country separately on data clustered in the various waves.

In the model, we used family affluence, family structure and year of survey as independent variables.

The estimation methods of pseudo-likelihood, binomial probability distribution and Logit link function were used. Only the countries with complete information and ≥50% of daily associations were selected for this analysis. This indicates that the predictive ability of the model to properly categorize who eats breakfast every day is higher than 50%. Otherwise, it would indicate that the model is unable to classify these subjects correctly and therefore the model would be poor. Estimate results were run for each country independently, with adolescents nested within classes and classes within schools (three-level random intercept model) and adjusted for age group. Bonferroni’s sequential test was applied to compare the DBC among the categories of under-consideration variables. Odds Ratios (ORs) and associated 95% Confidence Intervals (95% Cis) were calculated, and Wald’s test was applied to identify the relevant estimated parameters. The value of Bayesian Information Criterion (DIC) was used as a test measure of model fit. Fixed and non-fixed variable estimates of the model were tabulated, where the fixed estimates were defined as the mean effect on the entire population of schools, classes and adolescents, and the variable estimates described how these differed at each level (school and classes). All independent variables are presented as dummy indicator variables, contrasted against a base category Explanatory variables and their reference category: Family Affluence (Low), Family Structure (Single) and Survey Year (2018). *p*-values < 0.05 were considered significant. The ‘Generalized Linear Mixed Models’ in the statistical package SPSS (v.22.0) was used for all the analyses.

## 3. Results

Data on 589,737 adolescents in 23 countries or regions were included (Table 1). In total, boys and girls accounted for 48.8% and 51.2% of the sample population, respectively, with small differences noted between the countries. DBC ranged from 38.1% (Slovenia) to 72.1% (the Netherlands). In Central and Northern Europe, no country had DBC below 50%; in Southern Europe, DBC was 49.4% in Croatia and 38.1% in Slovenia; and in Eastern Europe, DBC was 47% in Czech Republic and 44% in Hungary.

Table 1 shows that the proportion of children living in high-affluence homes ranged from 7.9% (Ukraine) to 60.0% (Norway); the medium-affluence group ranged from 36.0% (Norway) to 56.6% (Spain); and the low-affluence group ranged from 3.5% (Norway) to 44.6% (Ukraine). For family structure, the proportion of children living with both parents ranged from 55.5% (Russian Federation) to 86.9% (Macedonia), while between 8.6% (Macedonia) and 29.4% (Scotland) of children lived in single-parent households.

As shown in Table 2, with the exception of a few countries and survey years (Macedonia, 2006–2018; Latvia 2010; Sweden 2014; Russian federation 2018; Ukraine 2016, 2018), females reported skipping daily breakfast more frequently than males. The lowest breakfast consumption frequencies were observed among Slovenian males (37.0%) and females (32.7%) in 2002; Slovenian males (37.6%) and females (36.6%) in 2006; Slovenian males (39.7%) and females (37.9%) in 2010; Macedonian males (25.2%) and females (26.3%) in 2014; and Slovenian males (39.6%) and Hungarian females (35.0%) in 2018. In contrast, the highest daily breakfast consumption frequencies were observed among Portuguese males (75.9%) and females (69.2%) in 2002; Portuguese males (72.2%) and Dutch females (68.7%) in 2006; Dutch males (76.4%) and females (75.0%) in 2010; Portuguese males (77.5%) and females (72.5%) in 2014; and Dutch males (75.7%) and females (69.3%) in 2018.

### 3.1. Family Affluence

The prevalence levels of country-specific DBC by family affluence scale (FAS), family structure and year of survey are presented in Table 3. The distribution of DBC by FAS showed that in the high-affluence group, DBC ranged from 37.3% (Slovenia) to 72.7% (the Netherlands); from 34.2% (Slovenia) to 65.8% (Portugal) in the medium-affluence group; and from 31.3% (Slovenia) to 61.2% (Netherlands) in the low-affluence group. The results of the logistic regression applied to daily breakfast consumption are shown in Table 4. In most countries (*n* = 19), DBC was positively linked to being an adolescent living in a family with high FAS compared to living in a family with low FAS. OR ranged from 1.18 in Croatia (95% CI: 1.09–1.27) to 2.09 (95% CI: 1.89–2.31) in Germany. In the Russian Federation and Latvia, no correlation between DBC and family affluence was found (Table 4).

### 3.2. Family Structure

Concerning adolescents’ family structure, the results highlighted that, in all the countries studied, adolescents living in two-parent families were more likely to report DBC compared to single-parent families. In most countries, the young people living in ‘other’ types of family structures reported daily breakfast consumption frequencies between the frequencies reported by children from two-parent and single-parent families. Specifically, DBC ranged from 37.1% (Slovenia) to 72.9% (the Netherlands) for adolescents living in two-parent families, while in single-parent families, the range was from 31.6% (Slovenia) to 63.1% (the Netherlands). DBC for youths living in ‘other’ family structures ranged from 34.1% (Slovenia) to 65.0% (the Netherlands). In the regression analysis, DBC was associated with being an adolescent of a two-parent family with an OR from 1.14 in Ukraine (95% CI: 1.06–1.20) to 1.72 (95% CI: 1.60–1.84) in the Netherlands, compared to being a child in a single-parent family (Table 4).

### 3.3. Trends in Daily Breakfast Consumption over Time

Distributions of DBC by wave revealed that in many countries the proportion of adolescents reporting DBC was lower in 2018 compared to 2002. In 2002, DBC ranged from 31.6% (Slovenia) to 68.1% (Latvia); in 2006, it ranged from 33.9% (Slovenia) to 65.9% (Portugal); in 2010, it ranged from 35.0% (Slovenia) to 70.4% (the Netherlands); in 2014, it ranged from 18.0% (Macedonia) to 70.3% (Portugal); and in 2018, it ranged from 37.2% (Hungary) to 67.0% (the Netherlands) (Table 3).

Two countries showed a significant increase in DBC (the Netherlands and Macedonia) from 2002 to 2018. A significant decrease in DBC was observed in 17 countries (Belgium-Fr, France, Germany, Croatia, Portugal, Spain, Hungary, Poland, Russian Federation, Ukraine, Denmark, Scotland, Finland, Latvia, Lithuania, Sweden and Norway), while in four countries no significant changes were observed (Czech Republic, England, Ireland and Norway) (Table 3).

## 4. Discussion

This study contributes to the existing literature by being the first study to compare the DBC trend data of 23 European countries while using the same protocol and methodology to collect the data. The results showed the frequency of DBC ranging from 37.8% to 72.6% and being more common among boys. A significant decrease in DBC from 2002 to 2018 was observed in 15 countries (Belgium-Fr, France, Germany, Croatia, Portugal, Spain, Hungary, Poland, Russian Federation, Ukraine, Denmark, Finland, Latvia, Lithuania and Sweden), while in 4 countries no significant change was observed (Czech Republic, Scotland, Ireland and Norway). In most countries (*n* = 19), DBC was higher among adolescents from high-affluence homes, which is in line with other research [32].

In all the countries analysed, adolescents living in two-parent households reported higher DBC than those in single-parent households.

This study updated previous HBSC work [23] and added new information by studying trends in DBC over nearly a decade in a multinational context using the same standardized methods. The existing literature on DBC changes over time mainly includes national and local-level studies [21,22,25]. To our knowledge, this is one of the few studies that analyzes trends in a large number of countries. The observed differences in temporal trends in DBC between countries are difficult to explain, but there may be some common factors that require further investigation.

It was highlighted that there has been an increase in the availability of foods outside of the home, especially in Western countries, and that this might have contributed to a decrease in breakfast consumption [33,34]. Poor food habits could be exacerbated by these changes in the food environment, and, over time, might further exacerbate the rates of obesity and diabetes. Furthermore, according to the literature in the field, we have found that in a majority of countries the proportion of adolescents consuming daily breakfast was generally higher in two-parent families [6,7,8,9,10,11,12,13] and among boys [5].

Several studies have highlighted that the family environment influences the dietary behaviours of young people [19,35,36,37]. Adolescents’ health behaviours are affected by their parents’ beliefs, actions and attitudes during their socialization, which occurs within the family unit [38]. Parental eating behaviours are positively associated with both unhealthy [39] and healthy [15,23,39,40,41,42,43,44,45,46,47,48] dietary behaviours among young people. In our analysis, differences in DBC were observed between boys and girls. This could indicate that family-related processes affecting adolescents’ DBC may be influenced by sex. Weight concerns among adolescent girls [49] may influence the differences between males’ and females’ DBC [2,3,50,51].

More research is required to investigate which countries report an increase in DBC over time and the related changes in policies, strategies and programmes. In particular, in a multi-level analytical design, it would be useful to describe and compare national features and guidelines and their implementation while taking into account cultural and regulatory practices. Future research should include other potential determinants, such as lack of knowledge about health and nutrition [52], time to eat or prepare breakfast [53] and the unavailability of foods for breakfast [2], all of which can affect daily breakfast consumption among adolescents. To be effective, strategies to promote daily breakfast consumption need to be informed by an understanding of the drivers of breakfast skipping across population sub-groups.

Intervention programmes at a national level to increase DBC can be implemented to understand which behavioural strategies contribute to increasing breakfast intake. These need to be evaluated and measured using longitudinal methods and objective measures of behavioural change.

### Limitations and Strengths

In the discussion of the results, some study limitations should be considered.

The first point is that comparing adolescents who eat breakfast every day with those who eat it less than daily could suggest that skipping breakfast even just one day could not have effects on their health [7]. However, ‘daily’ was chosen as a consistent habitual breakfast routine that is related to superior dietary quality and health. The HBSC study defines breakfast as having more than a glass of fruit juice or milk; thus, it is based on a frequency measure where no assessment of the nutritional quality of the meal is made, as details of what breakfast includes are not available. Another issue is related to the use of family affluence over time. The classification by the family affluence measure may not be uniform over time, and, further, misclassifications of less affluent families into more affluent groups may therefore increase over time. From this perspective, there is the risk that the patterns of social inequality are increasingly being underestimated or overestimated in each survey year.

The major strength of these analyses is that they are based on a large and representative sample of adolescents (almost 600,000 boys and girls) from 23 countries, where data were collected according to a standardized protocol.

## 5. Conclusions

The results from the present study suggest that DBC is not trending upwards, and that if nations want to take DBC seriously, they must implement strategies to increase DBC. Thus, DBC should be encouraged in each family and deserves special attention during the transition from childhood to adolescence, when young people are more vulnerable [18]. More efforts should be made to reduce social inequalities in breakfast consumption both in the family and school contexts. Previous intervention studies have shown that interventions focused on DBC can increase DBC [54,55,56]. It is important to promote school breakfast programmes that aim to ensure that all students have access to a nutritious breakfast and that have the goal of promoting engagement with learning and ultimately improving academic outcomes.

The overall evidence suggests the importance of exploring the causes of breakfast skipping—which likely differ between various populations subgroups—in order to improve strategies to promote breakfast consumption. Schools and educational systems have the opportunity to educate adolescents to provide environments that encourage healthy habits. With regards to nutrition, including breakfast consumption, this could extend beyond the meal service and health education or promotional activities focused on changing people’s perceptions of breakfast intake.

We want to point out the importance of trends and the need to continue monitoring adolescent diets and DBC—especially in light of recent political, social and economic events, such as the COVID-19 pandemic, war and inflation.

## Figures and Tables

**Table 1:** Country specific study populations by socio-demographic characteristics, daily breakfast consumption and survey year.

	Gender (%)		Family Affluence (%)			Family Structure (%)			Breakfast (%)	Survey Year (Absolute Frequency)					
Country	Boy	Girl	High	Medium	Low	Two	Single	Other	Daily	2002	2006	2010	2014	2018	Total
**Central European countries**															
Belgium—Fr	49.7	50.3	39.0	48.2	12.8	68.2	27.1	4.6	54.4	4323	4476	4012	5892	4020	22,723
France	49.7	50.3	40.3	49.3	10.4	71.4	23.6	5.0	58.0	8185	7155	6160	5691	9170	36,361
Germany	49.4	50.6	40.5	49.1	10.4	73.5	22.4	4.0	56.4	5650	7274	5005	5961	4347	28,237
Netherlands	49.3	50.7	48.6	46.0	5.4	79.1	19.4	1.6	72.1	4268	4278	4591	4301	4698	22,136
**Southern European countries**															
Croatia	49.6	50.4	20.5	54.7	24.8	84.5	11.8	3.7	49.4	4397	4968	6262	5741	5169	26,537
Macedonia	49.7	50.3	18.4	49.8	31.8	86.9	8.6	4.5	50.4	4161	5281	3944	4218	4658	22,262
Portugal	47.6	52.4	30.0	53.9	16.0	71.8	18.7	9.5	68.7	2940	3919	4036	4989	6126	22,010
Slovenia	50.2	49.8	44.8	46.8	8.4	82.0	15.1	2.9	38.1	3956	5130	5436	4997	5667	25,186
Spain	49.0	51.0	30.8	56.6	12.6	80.2	15.3	4.5	63.4	5827	8891	5040	11,136	4320	35,124
**Eastern European countries**															
Czech Republic	49.3	50.7	30.0	51.5	18.5	69.3	26.5	4.2	47.0	5012	4782	4425	5082	11,564	30,865
Hungary	47.3	52.7	21.2	51.0	27.8	72.1	24.8	3.1	44.0	4164	3532	4864	3935	3789	20,284
Poland	49.3	50.7	21.3	53.7	25.0	81.0	16.6	2.4	59.8	6383	5489	4262	4545	5224	25,903
Russian Federation	47.1	52.9	11.8	48.0	40.2	55.5	21.5	23.0	52.9	8037	8231	5174	4716	4281	30,439
Ukraine	47.6	52.4	7.9	47.5	44.6	73.2	23.8	3.0	60.8	4090	5069	5890	4552	6660	26,261
**Northern European countries**															
Denmark	48.0	52.0	46.4	46.6	7.0	65.1	25.4	9.5	66.9	4672	5741	4330	3891	3181	21,185
Scotland	49.6	50.4	42.0	47.4	10.6	65.5	29.4	5.2	51.6	4404	6190	6771	5932	5021	28,318
England	49.2	50.8	41.5	48.3	10.2	65.8	27.3	7.0	50.5	6081	4783	3524	5335	3397	23,120
Finland	49.0	51.0	35.2	55.1	9.7	73.0	24.5	2.5	60.8	5388	5249	6723	5925	3146	26,431
Ireland	48.1	51.9	27.5	58.2	14.3	75.1	18.2	6.7	60.2	2875	4894	4965	4098	3833	20,665
Latvia	48.2	51.8	22.6	49.8	27.6	64.0	31.3	4.7	58.5	3481	4245	4284	5557	4412	21,979
Lithuania	51.1	48.9	23.2	50.1	26.7	71.3	25.4	3.3	54.6	5645	5632	5338	5730	3797	26,142
Sweden	49.9	50.1	47.4	47.0	5.5	71.7	24.5	3.8	63.9	3926	4415	6718	7700	4185	26,944
Norway	50.2	49.8	60.0	36.6	3.5	68.9	21.9	9.2	62.2	5023	4711	4342	3422	3127	20,625

**Table 2:** Daily breakfast consumption by country, survey year and gender (%).

	2002		2006		2010		2014		2018	
Country	Male	Female	Male	Female	Male	Female	Male	Female	Male	Female
**Western European countries**										
Belgium—Fr	61.4	54.4	56.8	56.8	55.2	53.2	54.6	49.9	52.9	50.6
France	66.6	59.4	60.8	54.8	61.0	53.1	59.3	53.4	57.5	53.6
Germany	62.2	57.6	61.6	54.9	60.3	57.5	56.4	51.6	51.8	46.6
Netherlands	70.1	65.5	72.1	68.7	76.4	75.0	77.3	70.8	75.7	69.3
**Southern European countries**										
Croatia	67.0	62.1	52.7	48.1	51.2	48.0	45.4	43.9	42.9	38.6
Macedonia	52.9	50.5	54.4	56.7	55.0	60.2	25.2	26.3	58.1	61.2
Portugal	75.9	69.2	72.2	68.6	73.3	67.7	77.5	72.5	63.4	56.3
Slovenia	37.0	32.7	37.6	36.6	39.7	37.9	42.0	39.1	39.6	37.6
Spain	69.9	61.5	70.5	64.2	58.9	51.8	67.9	62.0	62.3	54.1
**Eastern European countries**										
Czech Republic	52.8	41.8	46.7	40.6	51.4	45.6	49.3	47.7	50.2	44.0
Hungary	51.7	43.0	47.3	41.2	46.5	41.4	49.3	42.7	43.8	35.0
Poland	68.7	63.0	64.0	57.5	57.8	53.7	60.9	56.2	58.8	53.2
Russian Fed.	65.5	60.3	53.4	48.6	53.3	48.5	50.0	44.3	46.6	47.9
Ukraine	71.3	68.7	60.8	55.8	60.2	56.8	60.4	61.9	58.1	60.0
**Northern European countries**										
Denmark	70.3	66.3	71.1	65.7	67.8	66.2	68.9	63.1	64.7	62.8
Scotland	58.7	45.3	54.5	46.5	56.8	48.0	56.6	48.5	54.4	47.3
England	52.4	41.0	59.8	49.8	50.1	44.1	61.8	47.5	53.2	44.1
Finland	65.4	55.7	60.9	57.0	62.5	60.9	65.6	61.6	58.3	55.9
Ireland	67.7	56.5	61.9	56.1	60.6	55.1	65.1	57.1	67.3	59.7
Latvia	70.2	68.1	60.7	58.2	56.6	57.1	59.4	55.9	54.3	50.3
Lithuania	70.3	62.4	59.5	54.7	51.3	49.1	52.0	49.8	45.9	43.9
Sweden	70.8	65.1	69.6	66.4	65.7	62.0	62.5	62.6	62.0	54.9
Norway	65.2	58.4	67.2	60.6	63.3	60.5	62.0	59.8	63.1	60.6

**Table 3:** Daily breakfast consumption by country, family affluence, family structure and survey year.

	Family Affluence (%)			Family Structure (%)			Survey Year (%)				
Country	High	Medium	Low	Two	Single	Other	2002	2006	2010	2014	2018
**Western European countries**											
Belgium—Fr	57.9 ^a^	48.7 ^b^	40.3 ^a,b^	54.1 ^c^	47.0 ^c^	45.7	55.0 ^d^	52.7 ^e^	48.9 ^f^	45.4 ^g^	42.6 ^d,e,f,g^
France	57.8 ^a^	54.7 ^b^	47.8 ^a,b^	58.4 ^c^	50.2 ^c^	51.7	59.5 ^d^	53.5 ^e^	51.7	52.0	50.5 ^d,e^
Germany	59.4 ^a^	51.7 ^b^	41.2 ^a,b^	55.9 ^c^	47.7 ^c^	48.8	58.1 ^d^	53.8 ^e^	52.9 ^f^	47.9 ^g^	41.3 ^d,e,f,g^
Netherlands	72.7 ^a^	67.1 ^b^	61.2 ^a,b^	72.9 ^c^	63.1 ^c^	65.0	62.5 ^d^	65.8	70.4 ^e^	69.7	67.0 ^d,e^
**Southern European countries**											
Croatia	47.8 ^a^	46.5 ^b^	43.8 ^a,b^	51.3 ^c^	47.5 ^c,d^	39.4 ^d^	60.9 ^e^	45.5 ^f^	44.9 ^g^	40.9 ^h^	37.9 ^e,f,g,h^
Macedonia	42.5	43.6 ^a^	41.3 ^a^	48.9 ^b^	39.9 ^b^	38.8	44.6 ^c^	48.2 ^d^	52.4	18.6 ^e^	53.7 ^c,d,e^
Portugal	67.3 ^a^	65.8	60.1 ^a,b^	72.3 ^c^	64.2 ^d^	56.1 ^c,d^	66.4 ^e^	65.9 ^f^	65.3 ^g^	70.3 ^h^	53.6 ^e,f,g,h^
Slovenia	37.3 ^a^	34.2 ^b^	31.3 ^a,b^	37.1 ^c^	31.6 ^c^	34.1	31.6	33.9	35.0	36.6	34.2
Spain	62.9 ^a^	59.9 ^b^	55.0 ^a,b^	63.8 ^c^	55.4 ^c^	58.5	62.2 ^d^	64.3 ^e^	52.9	62.0 ^f^	54.7 ^d,e,f^
**Eastern European countries**											
Czech Republic	47.7 ^a^	46.6 ^b^	41.1 ^a,b^	49.7 ^c^	40.1 ^c,d^	43.6 ^d^	45.8	42.0	45.7	45.3	43.5
Hungary	45.0 ^a^	43.2 ^b^	40.1 ^a,b^	45.1 ^c^	40.3 ^c^	42.9	45.7 ^d^	43.3 ^e^	42.8 ^f^	45.0 ^g^	37.2 ^d,e,f,g^
Poland	60.4 ^a^	57.6 ^b^	52.9 ^a,b^	61.0 ^c^	52.9 ^c^	57.0	64.1 ^d^	58.8 ^e^	52.4	56.4 ^f^	52.9 ^d,e,f^
Russian Feder.	52.2	51.3	51.0	53.1 ^a^	48.2 ^b^	53.2 ^a,b^	62.7 ^c^	51.0	48.6	47.1	47.9 ^c^
Ukraine	61.0	62.2 ^a^	59.0 ^a^	62.5 ^b^	59.5 ^b^	60.1	69.9 ^c^	57.3	57.4	60.6 ^d^	58.0 ^c,d^
**Northern European countries**											
Denmark	68.0 ^a^	62.9 ^b^	54.4 ^a,b^	66.5 ^c^	57.4 ^c,d^	61.6 ^d^	65.7 ^e^	64.3 ^f^	63.0 ^g^	60.5 ^h^	55.7 ^e,f,g,h^
Scotland	53.3 ^a^	48.8 ^b^	43.0 ^a,b^	53.4 ^c^	43.6 ^c,d^	48.1 ^d^	48.4 ^e^	49.3 ^f^	49.5 ^g^	48.9 ^h^	45.6 ^e,f,g,h^
England	49.8 ^a^	45.4 ^b^	40.0 ^a,b^	52.7 ^c^	42.0 ^c^	40.5	41.1	49.0 ^d^	42.8	48.8 ^e^	43.7 ^d,e^
Finland	59.0 ^a^	57.3 ^b^	53.5 ^a,b^	62.8 ^c^	51.1 ^c,d^	55.8 ^d^	57.1 ^e^	55.5 ^f^	57.4 ^g^	59.9 ^h^	53.1 ^e,f,g,h^
Ireland	61.0 ^a^	58.3 ^b^	51.1 ^a,b^	63.1 ^c^	50.9 ^c,d^	56.4 ^d^	56.3	56.2	56.0	57.1	58.6
Latvia	58.4	58.5	58.3	61.1 ^a^	55.3 ^a^	58.7	68.1 ^b^	58.7 ^c^	56.4 ^d^	56.5 ^e^	51.6 ^b,c,d,e^
Lithuania	54.7 ^a^	53.3 ^b^	47.4 ^a,b^	54.8 ^c^	48.5 ^c^	52.1	64.5 ^d^	56.1 ^e^	48.2 ^f^	48.1 ^g^	41.7 ^d,e,f,g^
Sweden	63.1 ^a^	59.2 ^b^	49.1 ^a,b^	64.8 ^c^	55.2 ^c,d^	51.4 ^d^	61.5 ^e^	61.0 ^f^	56.0 ^g^	55.6 ^h^	51.7 ^e,f,g,h^
Norway	59.8 ^a^	53.6 ^b^	44.0 ^a,b^	61.5 ^c^	48.2 ^c^	47.6	54.2 ^d^	55.5 ^e^	50.9	51.2	50.7 ^d,e^

Note. Bonferroni sequential test, *p* < 0.05. The same superscript (^a,b,c,d,e,f,g,h^) in the rows for each variable identifies a significant difference.

**Table 4:** Associations of daily breakfast consumption with family affluence, family structure and survey year with daily breakfast consumption by country.

	Family Affluence (OR [95% CI])		Family Structure (OR [95% CI])		Survey Year (OR [95% CI])			
Country	High	Medium	Two	Other	2002	2006	2010	2014
**Central European countries**								
Belgium—Fr	**2.04 (1.86–2.24)**	**1.41 (1.29–1.54)**	**1.33 (1.25–1.42)**	0.95 (0.82–1.10)	**1.65 (1.48–1.83)**	**1.50 (1.35–1.67)**	**1.29 (1.16–1.43)**	**1.12 (1.01–1.23)**
France	**1.50 (1.39–1.62)**	**1.32 (1.22–1.42)**	**1.39 (1.32–1.46)**	1.06 (0.95–1.18)	**1.44 (1.34–1.55)**	**1.13 (1.05–1.21)**	1.05 (0.98–1.14)	1.06 (0.98–1.15)
Germany	**2.09 (1.89–2.31)**	**1.53 (1.38–1.68)**	**1.39 (1.30–1.48)**	1.04 (0.90–1.21)	**1.97 (1.70–2.29)**	**1.65 (1.50–1.82)**	**1.60 (1.45–1.77)**	**1.31 (1.19–1.44)**
Netherlands	**1.69 (1.47–1.93)**	**1.29 (1.13–1.47)**	**1.57 (1.46–1.70)**	1.08 (0.85–1.38)	**0.82 (0.73–0.92)**	0.95 (0.85–1.06)	**1.17 (1.05–1.32)**	**1.14 (1.01–1.27)**
**Southern European countries**								
Croatia	**1.18 (1.09–1.27)**	**1.12 (1.05–1.19)**	**1.16 (1.08–1.26)**	**0.72 (0.61–0.85)**	**2.56 (2.29–2.85)**	**1.37 (1.23–1.52)**	**1.34 (1.22–1.47)**	**1.14 (1.03–1.26)**
Macedonia	1.05 (0.96–1.15)	**1.10 (1.03–1.18)**	**1.44 (1.29–1.60)**	0.96 (0.80–1.15)	**0.70 (0.57–0.85)**	**0.80 (0.67–0.97)**	0.95 (0.79–1.15)	**0.20 (0.16–0.24)**
Portugal	**1.37 (1.24–1.51)**	**1.28 (1.17–1.39)**	**1.45 (1.35–1.57)**	**0.71 (0.63–0.81)**	**1.72 (1.53–1.92)**	**1.68 (1.51–1.86)**	**1.63 (1.47–1.81)**	**2.05 (1.86–2.28)**
Slovenia	**1.31 (1.18–1.46)**	**1.14 (1.03–1.27)**	**1.28 (1.18–1.38)**	1.12 (0.94–1.34)	**0.89 (0.80–0.99)**	0.99 (0.89–1.09)	1.04 (0.94–1.14)	**1.11 (1.01–1.23)**
Spain	**1.39 (1.28–1.50)**	**1.22 (1.14–1.32)**	**1.42 (1.33–1.51)**	1.13 (0.99–1.30)	**1.36 (1.24–1.49)**	**1.49 (1.37–1.62)**	0.93 (0.84–1.02)	**1.35 (1.24–1.47)**
**Eastern European countries**								
Czech Republic	**1.31 (1.22–1.41)**	**1.16 (1.09–1.24)**	**1.48 (1.40–1.56)**	**1.15 (1.02–1.30)**	**1.10 (1.01–1.19)**	0.94 (0.87–1.02)	**1.09 (1.01–1.18)**	1.07 (1.00–1.15)
Hungary	**1.22 (1.12–1.33)**	**1.14 (1.06–1.22)**	**1.22 (1.14–1.30)**	1.12 (0.94–1.33)	**1.42 (1.27–1.59)**	**1.29 (1.15–1.45)**	**1.27 (1.14–1.41)**	**1.38 (1.24–1.54)**
Poland	**1.36 (1.26–1.47)**	**1.21 (1.14–1.29)**	**1.39 (1.30–1.49)**	1.18 (0.99–1.41)	**1.59 (1.45–1.75)**	**1.28 (1.16–1.40)**	0.98 (0.89–1.08)	**1.15 (1.05–1.27)**
Russian Feder.	1.05 (0.97–1.14)	1.01 (0.96–1.07)	**1.22 (1.15–1.29)**	**1.22 (1.07–1.40)**	**1.83 (1.65–2.02)**	**1.13 (1.02–1.26)**	1.03 (0.87–1.21)	0.97 (0.87–1.08)
Ukraine	1.09 (0.98–1.20)	**1.15 (1.09–1.21)**	**1.14 (1.07–1.21)**	1.03 (0.88–1.20)	**1.66 (1.51–1.83)**	0.97 (0.89–1.06)	0.98 (0.90–1.06)	**1.12 (1.02–1.22)**
**Northern European countries**								
Denmark	**1.78 (1.58–2.01)**	**1.42 (1.27–1.60)**	**1.48 (1.38–158)**	**1.19 (1.06–1.34)**	**1.52 (1.35–1.71)**	**1.43 (1.27–1.61)**	**1.36 (1.20–1.53)**	**1.22 (1.05–1.41)**
Scotland	**1.51 (1.38–1.65)**	**1.27 (1.16–1.38)**	**1.49 (1.41–1.57)**	**1.20 (1.06–1.37)**	**1.12 (1.01–1.24)**	**1.16 (1.06–1.27)**	**1.17 (1.07–1.28)**	**1.14 (1.04–1.25)**
England	**1.49 (1.34–1.65)**	**1.24 (1.13–1.37)**	**1.53 (1.44–1.64)**	0.94 (0.82–1.08)	0.90 (0.80–1.01)	**1.24 (1.10–1.40)**	0.97 (0.85–1.10)	**1.23 (1.09–1.39)**
Finland	**1.25 (1.13–1.38)**	**1.17 (1.07–1.28)**	**1.62 (1.52–1.72)**	**1.21 (1.01–1.45)**	**1.17 (1.05–1.31)**	1.10 (0.99–1.23)	**1.19 (1.07–1.32)**	**1.32 (1.19–1.46)**
Ireland	**1.50 (1.35–1.66)**	**1.38 (1.22–1.46)**	**1.65 (1.52–1.78)**	**1.24 (1.07–1.45)**	0.91 (0.81–1.03)	0.91 (0.81–1.01)	0.90 (0.80–1.01)	0.94 (0.84–1.05)
Latvia	1.01 (0.93–1.09)	1.01 (0.94–1.08)	**1.27 (1.20–1.35)**	1.15 (1.00–1.32)	**2.01 (1.80–2.24)**	**1.34 (1.21–1.48)**	**1.22 (1.10–1.34)**	**1.22 (1.11–1.34)**
Lithuania	**1.34 (1.24–1.45)**	**1.27 (1.19–1.35)**	**1.29 (1.22–1.37)**	1.16 (1.00–1.34)	**2.54 (2.19–2.95)**	**1.79 (1.62–1.99)**	**1.30 (1.18–1.44)**	**1.30 (1.17–1.43)**
Sweden	**1.77 (1.58–1.99)**	**1.50 (1.34–1.69)**	**1.49 (1.41–1.59)**	**0.86 (0.74–0.99)**	**1.49 (1.34–1.66)**	**1.46 (1.32–1.62)**	**1.19 (1.09–1.30)**	**1.17 (1.08–1.27)**
Norway	**1.89 (1.61–2.22)**	**1.47 (1.25–1.73)**	**1.72 (1.60–1.84)**	0.97 (0.87–1.09)	**1.15 (1.03–1.28)**	**1.22 (1.09–1.36)**	1.01 (0.90–1.13)	1.02 (0.91–1.15)

Reference category: Family Affluence (Low), Family Structure (Single) and Survey Year (2018). For each variable, the missing category is the reference. Wald test, *p* < 0.05 Adjusted by random effects of school and school class; Association daily-daily < 50%: Slovenia, Czech Republic, Hungary; adjusted for Family Affluence, Family Structure and Survey Year. In Bold are reported the statistically significant results.

## Data Availability

HBSC data and questionnaires can be accessed via a request to the HBSC Data Management Centre: dmc@hbsc.org. For further information, see http://www.uib.no/en/hbscdata (accessed on 25 April 2023).
